# Metformin Alters Gut Microbiota of Healthy Mice: Implication for Its Potential Role in Gut Microbiota Homeostasis

**DOI:** 10.3389/fmicb.2018.01336

**Published:** 2018-06-22

**Authors:** Wei Ma, Ji Chen, Yuhong Meng, Jichun Yang, Qinghua Cui, Yuan Zhou

**Affiliations:** ^1^Department of Biomedical Informatics, School of Basic Medical Sciences, Peking University, Beijing, China; ^2^Ministry of Education Key Laboratory of Molecular Cardiovascular Sciences, Peking University, Beijing, China; ^3^Central Laboratory, PLA Navy General Hospital, Beijing, China; ^4^Department of Physiology and Pathophysiology, School of Basic Medical Sciences, Peking University, Beijing, China

**Keywords:** metformin, gut microbiome, diabetes, 16S rRNA sequencing, MicroPattern

## Abstract

In recent years, the first-line anti-diabetic drug metformin has been shown to be also useful for the treatment of other diseases like cancer. To date, few reports were about the impact of metformin on gut microbiota. To fully understand the mechanism of action of metformin in treating diseases other than diabetes, it is especially important to investigate the impact of long-term metformin treatment on the gut microbiome in non-diabetic status. In this study, we treated healthy mice with metformin for 30 days, and observed 46 significantly changed gut microbes by using the 16S rRNA-based microbiome profiling technique. We found that microbes from the *Verrucomicrobiaceae* and *Prevotellaceae* classes were enriched, while those from *Lachnospiraceae* and *Rhodobacteraceae* were depleted. We further compared the altered microbiome profile with the profiles under various disease conditions using our recently developed comparative microbiome tool known as MicroPattern. Interestingly, the treatment of diabetes patients with metformin positively correlates with colon cancer and type 1 diabetes, indicating a confounding effect on the gut microbiome in patients with diabetes. However, the treatment of healthy mice with metformin exhibits a negative correlation with multiple inflammatory diseases, indicating a protective anti-inflammatory role of metformin in non-diabetes status. This result underscores the potential effect of metformin on gut microbiome homeostasis, which may contribute to the treatment of non-diabetic diseases.

## Introduction

Metformin, also known as 1,1-dimethylbiguanide has been widely used in the treatment of type 2 diabetes mellitus (T2DM) since 1958 in United Kingdom and 1995 in United States (Witters, 2001). The main mechanisms underlying its anti-hyperglycemia effect include decreasing intestinal absorption of glucose, increasing insulin sensitivity and decreasing hepatic glucose production (Bailey and Turner, 1996; Hundal et al., 2000), which together result in reduction of basal and postprandial glucose levels. Because clinical investigations have shown that metformin has low risk of hypoglycemia, modest weight loss, persistent anti-hyperglycemic effect and cardiovascular safety, it is now approved as one of the first-line drugs for treating T2DM (Garber et al., 2013).

Interestingly, it was shown in 2005 that metformin has the anti-cancer properties (Evans et al., 2005). Then a series of studies provided supporting evidence of its anti-cancer effects in a variety of cancer types such as ovarian cancer (Shank et al., 2012), endometrial cancer (Shafiee et al., 2014), breast cancer (Zhang and Li, 2014), liver cancer, pancreatic cancer, esophageal cancer, gastric cancer, and colorectal cancer (Franciosi et al., 2013). Metformin has been shown to reduce the incidence and mortality of cancer and block migration and invasion of tumor cells (Bao et al., 2012; Wu et al., 2015). Current knowledge pertaining to the molecular mechanisms underlying the anti-cancer activity of metformin is focused on two pathways that inhibit mTOR: (1) the AMPK-dependent pathway, in which metformin activates LKB1-AMPK to inhibit mTOR; (2) the AMPK-independent pathway, in which metformin inhibits mTOR via the PI3K/Akt/mTOR cascade (Sosnicki et al., 2016; Zhang and Guo, 2016). Besides, many studies have shown that metformin could also be utilized for the treatment of other diseases including obesity, polycystic ovary syndrome and tuberculosis (Gundelach et al., 2016; Igel et al., 2016; Restrepo, 2016). Moreover, it was reported that metformin also has potentially anti-aging effects (Novelle et al., 2016).

Body microbiota play extensive roles in physiology and it is therefore known as a “forgotten organ” (O’Hara and Shanahan, 2006). A lot of diseases are associated with gut microbiota, including cancer (Schwabe and Jobin, 2013; May et al., 2016), cardiovascular diseases (Koeth et al., 2013; Tang et al., 2013), obesity (Ley, 2010), diabetes (Wen et al., 2008; Qin et al., 2012), multiple sclerosis (Ezendam et al., 2008; Berer et al., 2011; Lee et al., 2011), neuromyelitis optica (Varrin-Doyer et al., 2012; Banati et al., 2013), Guillain–Barré syndrome (Ochoa-Reparaz et al., 2011), central nervous system disorders (Wang and Kasper, 2014) and autoinflammatory diseases (Lukens et al., 2014). For example, in obese individuals, *Bacteroidetes* is decreased whereas *Firmicutes* is increased (Furet et al., 2010), while *Prevotellaceae* that could produce H_2_ is increased and methanogenic archaea which could utilize H_2_ is also increased (Zhang et al., 2009). The co-existence of H_2_-producing bacteria with relatively high numbers of H_2_-utilizing methanogenic archaea in the gastrointestinal tract of obese individuals implies the plausible inter-species H_2_ transfer between bacterial and archaeal species as an important mechanism for increasing intestinal energy uptake in obese persons. Moreover, in the gut of human subjects with type 2 diabetes, *Firmicutes* is significantly decreased (Larsen et al., 2010). The abundance of *Faecalibacterium prausnitzii* was negatively correlated with both diabetic and inflammatory markers, which indicated that *Faecalibacterium prausnitzii* could regulate inflammation in gut in diabetic patients (Furet et al., 2010). Finally, Bolte et al. found that in gut of autistic disorder, *Clostridium tetani* is increased while Finegold et al. found that *Bacteroidetes* is also increased. (Bolte, 1998; Finegold et al., 2010). Since the gut microbiota play important roles in the development of various diseases, they are intuitively one of the first targets for drugs and may also contribute to the effect of metformin in treating diseases like cancer and T2DM. Nevertheless, to date, few studies have taken gut microbiota into consideration. As a result, current knowledge about the mechanism of action of metformin is still not completed. Since metformin has been suggested to treat a wide spectrum of diseases other than T2DM even for healthy individuals, it is especially important to assess the effect of metformin on the gut microbiota considering its potential long-term usage in healthy conditions. What microbes are regulated by metformin in healthy individuals? How do these regulated microbes associate with other diseases? What is the difference of changes of gut microbiota between healthy and various diseases when treated with metformin? A profiling of the gut microbiome under healthy condition is necessary to answer these questions.

In this study, we treated healthy mice with metformin for 30 days and used 16S rRNA sequencing to evaluate the abundance of microbes in fecal samples. By comparing the metformin-treated healthy mice to the mock controls, we observed 46 significantly changed microbes. In addition, from previous publications, we also obtained significantly changed microbes from T2DM patients after metformin treatment (Forslund et al., 2015; Wu et al., 2017; Allin et al., 2018). We then used MicroPattern, a tool we recently developed for the comparison of microbiome profiles under different situations, to analyze these significantly altered microbes. By procedure, whether metformin could elicit different alterations of gut microbiota under diabetes and non-diabetic conditions, respectively, were evaluated and discussed.

## Materials and Methods

### Animal Protocol and Sample Collection

This study was carried out in accordance with the principles of the Basel Declaration and recommendations of the Guide for the Care and Use of Laboratory Animals, US National Institutes of Health (NIH Publication No. 85-23, revised 1996). The protocol was approved by the Animal Research Committee of the Peking University Health Science Center. More specifically, 19 C57BL/6 healthy mice were separated into two groups: 9 were controls and 10 were included in the metformin-treated group. Until 8 weeks of age, mice were maintained on a chow diet. Then, mice in the metformin-treated group were treated with metformin (300 mg/kg of body weight) once daily via intragastric administration for 30 days. Mice in the control group were treated with an equivalent amount of saline via intragastric administration for 30 days. Fecal samples were obtained from 19 mice under sterile conditions. Because the microbiome profiling technique requires a large amount of raw materials, fecal was collected from 3 or 4 mice per sample (e.g., the fecal from the first, second and third mice was gathered together as the first sample). Finally, we acquired 3 metformin-treated samples and 3 mock control samples. Every sample was stored in a sterile 1.5 ml centrifuge tube at -80°C until microbiome profiling analysis.

### 16S rRNA Gene Sequencing

Microbial DNA was extracted from the fecal samples and the 16S rRNA gene of the isolated DNA was sequenced using Illumina Miseq2500 platform (service provided by GENE DENOVO Corporation) following the manufacturer’s guidelines. 16S rRNA gene sequences de-multiplexing, quality control and operational taxonomic unit (OTU) binning were performed using Mothur version 1.3.4.0 with the standard pipeline (Schloss et al., 2009). The statistical test was performed in R, a free tool for scientific computing. OTU pathway analysis was performed using the Phylogenetic Investigation of Communities by Reconstruction of Unobserved States (PICRUSt) tool (Langille et al., 2013).

### Enrichment Analysis and Disease Similarity Calculation

Enrichment analysis and disease similarity calculation were performed using MicroPattern (Ma et al., 2017). We just keep the significantly changed microbes at genus or species level as the input. At last, we acquired 46 microbes that changed significantly in metformin treated mice versus mock controls. To analyze the effect of metformin more comprehensively, we used the published human gut microbiome profiles under different diabetes situation, with or without metformin treatment (Forslund et al., 2015; Wu et al., 2017; Allin et al., 2018). First, Forslund et al. (2015) studied the effects of type 2 diabetes and metformin on the human gut microbiota. In their research, there are four group including healthy controls, type 1 diabetes mellitus (T1DM) patients, T2DM patients and metformin treated T2DM patients (MTT2DM). We acquired 36 significantly changed microbes from metformin treated T2DM patients contrasted against T2DM patients without metformin treatment, 26 significantly changed microbes from T2DM contrasted against healthy and 9 significantly changed microbes from T1DM patients contrasted against healthy controls. Second, Allin et al. (2018) studied the aberrant intestinal microbiota in individuals with prediabetes and 5 significantly changed microbes in prediabetes individuals versus healthy controls were obtained. Third, we got 29 significantly changed microbes from Wu et al.’s study about alteration of gut microbiome in treatment-naive T2DM patients after metformin treatment (Wu et al., 2017). We integrated those data and our data together for the comparison analysis. Finally, the significance of the similarity in microbiota changes was evaluated by permutation-based resampling test. More specifically, we shuffled the de-regulated microbiota between different diseases and re-calculated the similarity scores based on the randomly permutated microbiota. This procedure was repeated for 10000 times. For the observed positive similarity, if no higher similarity could be observed in more than 9000 out of 10000 such permutation tests, this similarity was considered significant. Likewise, for the observed negative similarity, if no lower similarity could be observed in more than 9000 out of 10000 such permutation tests, this similarity was considered significant. Such threshold also corresponded to a false discovery rate (FDR) threshold of 0.1.

## Results

### Effect of Metformin of Mice Gut Microbiota

**FIGURE 1:**
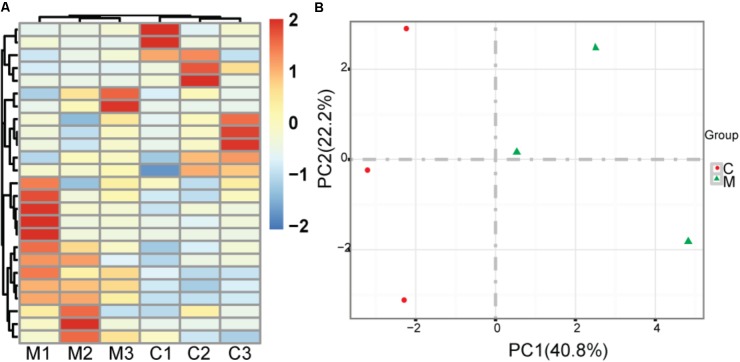
Effect of metformin on the gut microbiome in healthy mice. **(A)** Heatmap of metformin treated mice and mock controls based on top 25 abundant microbes at the genus level; M: metformin treated mice; C: mock controls. **(B)** Principal component analysis of metformin treated mice and mock controls, red rounds indicate mock controls and green triangles indicate metformin treated mice; PC1: the first principal component; PC2: the second principal component.

We treated healthy mice with metformin for 30 days and acquired significantly changed microbes in comparison with saline treated mock controls. To reduce noise, we just used the microbes whose tags occupy at least 0.1% of all tags. There is no significant difference of bacterial diversity between two groups with respect to Shannon diversity metrics (6.95 versus 6.87 for mean Shannon diversity of control group and metformin treated group, respectively, two sided *t*-test, *p* = 0.84) (Kemp and Aller, 2004). There are 46 significantly changed microbes, including 22 enriched microbes and 24 depleted microbes identified. At the class level, *Verrucomicrobiaceae*, *Prevotellaceae*, *Porphyromonadaceae*, *Rikenellaceae* are increased, while *Lachnospiraceae*, *Rhodobacteraceae* are decreased. Hierarchical clustering shows that samples from each group are clustered together (**Figure 1A**). Principal component analysis (PCA) suggests that metformin treated group and control group could be clearly separated in PC1, which explains 40.8% of the variation (**Figure 1B**). These results indicate that metformin consistently alters the gut microbiome of healthy mice.

**FIGURE 2:**
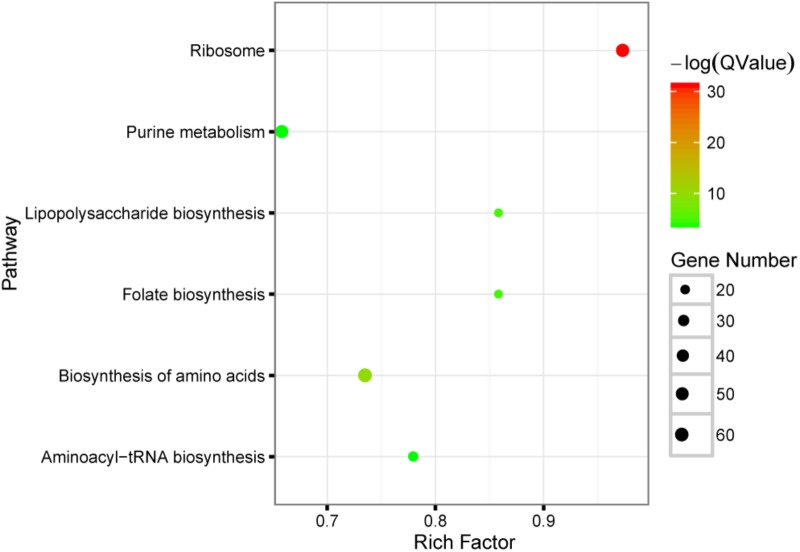
KEGG pathway enrichment analysis of the significantly changed microbes with metformin treatment. The result shown in this bubble plot was generated by using PICRUSt tool. Briefly, each microbe was assigned to several specific metabolic gene signatures, and whether these genes were enriched in certain pathway was tested. The size of bubble plot is correlated with the number of the genes presented in the specific pathways while the color conforms to the statistical significance of the pathway enrichment; Rich factor: the number of significantly changed genes divided by the total number of genes in one pathway.

To probe the function of these significantly changed microbes, PICRUSt was used to perform KEGG pathway analysis. Six pathways including ribosome, biosynthesis of amino acids, lipopolysaccharide biosynthesis, folate biosynthesis, purine metabolism and aminoacyl-tRNA biosynthesis are significant enriched (FDR < 0.05), see also **Figure 2**. The main function of gut microbiota is its important roles in metabolism, such as vitamin metabolism, short chain fatty acid metabolism, neuropeptide response, food digestion and so on. From the results of KEGG pathway analysis, it can be found that metformin mainly affects the gut microbiota related to such biological synthesis functions, including biosynthesis of lipopolysaccharide, folate, amino acids and proteins.

### Comparative Analysis of the Altered Microbiome Profile

**FIGURE 3:**
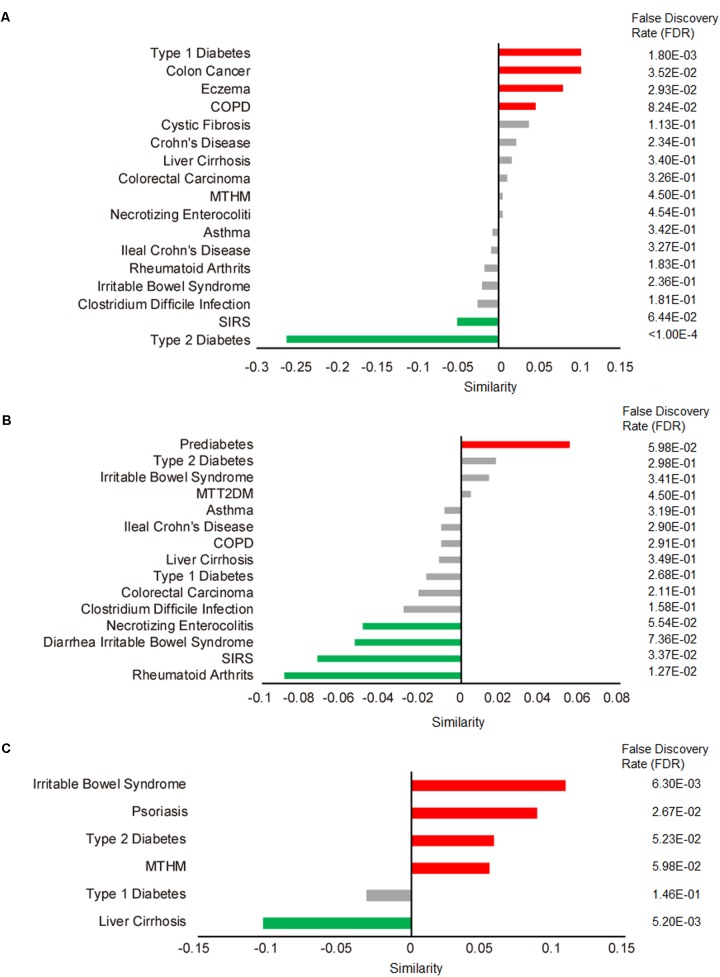
Correlated microbiome profile changes between metformin treatment and disease conditions. Red bars indicate significant positive similarities (FDR < 0.1 by 10000 times of permutation-based resampling test), while green bar indicate significant negative similarities. Insignificant similarities are shown as gray bars. The FDR is listed in details on the right side. **(A)** Microbe profile similarity between metformin treated T2DM (MTT2DM) and other diseases. COPD, chronic obstructive pulmonary disease; SIRS, systemic inflammatory response syndrome. **(B)** Microbe profile similarity between metformin treated healthy mice (MTHM) and other disease. **(C)** Microbe profile similarity between prediabetes and other disease.

We applied our comparative microbiome tool MicroPattern to compare the significantly changed microbiome profile by metformin treatment, with the de-regulated microbiome profiles under various disease situations. Thirty-six and 29 significantly changed microbes of metformin treated T2DM patients (MTT2DM), from Forslund et al. and Wu et al.’s studies respectively, were integrated together for the analysis. We first calculated microbiome similarity between (MTT2DM) patients and other diseases. Then we calculated microbiome similarity between metformin treated healthy mice (MTHM) and other diseases. Finally, we also calculated microbiome similarity between prediabetes and other diseases. There are 17 diseases exhibit microbiome profile similarity with MTT2DM. Among them, 5 of them are significant (FDR < 0.1), see **Figure 3A**. As intuitively expected, we found that the metformin treated T2DM has the largest negative similarity (-0.26, FDR < 1.00E-5) with T2DM. Metformin may reverse the changed microbes under T2DM and thus relieves diabetic conditions. In contrast, there are 15 diseases that exhibit microbiome profile similarity with MTHM. Among them, 4 of them show positive similarities while 11 show negative similarities (**Figure 3B**). In all, five significant diseases are observed and 4 of them show negative similarity, indicating that metformin may play an important role in gut microbiota homeostasis by reversing the disease-associated microbiome alteration. Finally, as for prediabetes, there are 6 diseases exhibiting microbiome profile similarity (**Figure 3C**). In the original study, the authors investigated the aberrant of intestinal microbiota in individuals with prediabetes, overweight, insulin resistance, dyslipidaemia and low-grade inflammation, which were precursors of T2DM. Interestingly, the similarity between T2DM and prediabetes is 0.058, which suggests that the gut microbiota plays an important role in T2DM pre-conditioning. We also performed the enrichment analysis to identify the associated disease situations for the significantly changed microbes in MTT2DM, MTHM and prediabetes groups. No term is enriched in MTT2DM, rheumatoid arthritis and colorectal carcinoma are enriched in MTHM, whereas liver cirrhosis and irritable bowel syndrome are enriched in prediabetes (**Table 1**). From this result, MTHM show strong association with colorectal carcinoma, suggesting that the effect of anti-colorectal carcinoma of metformin may at least partly be mediated via gut microbiota. Moreover, the prediabetes individuals show altered microbiota profile similar to that in irritable bowel syndrome (**Figure 3B**), while MTHM negatively correlates with diarrhea irritable bowel syndrome in terms of microbiota alteration. Therefore, the metformin treatment may also be beneficial to the gut microbiota homeostasis as it can partly act against the microbiota de-regulation in prediabetes. Finally, MTHM also has negative similarity with multiple inflammatory diseases such as diarrhea irritable bowel syndrome, necrotizing enterocolitis, systemic inflammatory response syndrome (SIRS) and rheumatoid arthritis, which collectively indicates that the anti-inflammatory role of metformin is also related to gut microbiota.

**Table 1:** MicroPattern disease enrichment analysis result.

Microbe set	*P*-value	FDR
**MTHM**		
Colorectal neoplasms	0.016	0.16
Arthritis, rheumatoid	0.016	0.16
**Prediabetes**		
Liver cirrhosis	0.0077	0.086
Irritable bowel syndrome	0.033	0.18

## Discussion

Metformin was found useful for its anti-T2DM, anti-cancer, anti-aging effects and the treatment of polycystic ovary syndrome (Gundelach et al., 2016; Heckman-Stoddard et al., 2016; Kedia et al., 2016; Novelle et al., 2016). Previous researches have shown that gut microbiota alterations may be partly responsible for metformin’s therapeutic effects against T2DM. For example, in diabetic rats, intravenous administration of metformin is less effective than intra-duodenal administration for lowering blood glucose levels (Bonora et al., 1984; Stepensky et al., 2002). Delayed-release metformin has lower bioavailability, and tends to accumulate in the lower bowel at higher concentrations compared with the common formulation (Stepensky et al., 2002; Buse et al., 2016). Changes of gut microbiota composition have been found in several diseases such as colon cancer (Wu et al., 2009; Kostic et al., 2013), rheumatoid arthritis (Scher et al., 2013), cardiovascular diseases (Wang et al., 2011; Tang et al., 2013) and diabetes (Larsen et al., 2010; Qin et al., 2012; Karlsson et al., 2013; Forslund et al., 2015; de la Cuesta-Zuluaga et al., 2017; Wu et al., 2017), obesity (Lee and Ko, 2014; Shin et al., 2014; Zhang et al., 2015). However, many questions exist. Could metformin alter gut microbiota of healthy individuals? How does metformin alter the gut microbiota of healthy individuals? What is the difference of the influence of metformin on gut microbiota under healthy and disease conditions? Does any correlation of microbiota alteration exist between different metformin treatment context? In our study, we treated healthy mice with metformin and found that metformin could indeed prominently affect gut microbiota under healthy condition. Subsequently, a computational method was applied for calculating the similarities between different conditions based on the changed microbes. Interestingly, the effects of metformin on gut microbiota turn out to be not identical under healthy and diabetes conditions. On the one hand, metformin could reverse the change of gut microbiota under diabetes, but the metformin treated mice did not show such trend. In fact, our result indicates a significant positive correlation with prediabetes and a weak positive correlation with T2DM. Therefore, although metformin shows a beneficial effect on gut microbiota in terms of improving disease condition in diabetic patients, our result cannot support the idea that metformin treatment of healthy mice could prevent diabetes-related gut microbiota disorder. To the contrary, metformin treatment of healthy mice may induce at least prediabetes. On the other hand, metformin treatment of diabetes patients positively correlates with colon cancer while metformin treatment of healthy mice exhibits negative correlation with multiple inflammatory diseases including diarrhea irritable bowel syndrome. This result indicates that metformin has potentially anti-inflammatory role especially under healthy condition. Indeed, the anti-inflammatory role of metformin was reported in previous research. For example, Koh et al. (2014) studied the anti-inflammatory mechanism of metformin but the proposed mechanism did not take gut microbiota into consideration. In their study, they found that metformin significantly inhibits interleukin (IL)-8 induction in COLO-205 cell stimulated with tumor necrosis factor (TNF)-α. Metformin significantly attenuates the severity of colitis in *IL-10*^-/-^ mice and inhibits the development of colitic cancer in mice. Similarly, in Liu et al.’s study, metformin significantly decreases the mRNA expression of *IL-6* and *THF-α* and increases the mRNA expression of PI3K and Akt in pancreatic tissue of T2DM rats. A lot of microbes are shown to be correlated with inflammatory factors such as IL-6 and THF-α (Liu et al., 2018). In Lee et al. (2017) study, *IL-1β* and *IL-6* expression was significantly decreased in metformin-treated in aged obese mice and *IL-1β* and *IL-6* expression is negatively correlated with the abundance of *Bacteroides*, *Butyricimonas*, *Anaerotruncus* and *Akkermansia*. These studies link the anti-inflammatory mechanism of metformin with gut microbes in disease conditions. Our results also indicate that gut microbiota may play an important role in the anti-inflammatory effect of metformin in non-diabetic condition, which complements the conclusions of the previous studies. In summary, our microbiome profiling analysis signifies the role of gut microbiome in the mechanism underlying metformin treatment, which deserves detailed experimental and clinical investigation in the future.

## Data Availability Statement

The 16S rRNA sequencing datasets generated in this study can be found in the SRA database (https://www.ncbi.nlm.nih.gov/sra/?term=SRP099828).

## Author Contributions

WM performed the computational analysis. JC and YM performed the animal experiments. WM and YZ drafted the manuscript. QC, YZ, and JY conceived and designed the study. QC and YZ supervised the study.

## Conflict of Interest Statement

The authors declare that the research was conducted in the absence of any commercial or financial relationships that could be construed as a potential conflict of interest.
